# Structural Analyses on the Deamidation of N-Terminal Asn in the Human N-Degron Pathway

**DOI:** 10.3390/biom10010163

**Published:** 2020-01-20

**Authors:** Joon Sung Park, Jae-Young Lee, Yen Thi Kim Nguyen, Nae-Won Kang, Eun Kyung Oh, Dong Man Jang, Hyun-Jung Kim, Dae-Duk Kim, Byung Woo Han

**Affiliations:** 1 Research Institute of Pharmaceutical Sciences, College of Pharmacy, Seoul National University, Seoul 08826, Korea; wingpjs@snu.ac.kr (J.S.P.); kimyen@snu.ac.kr (Y.T.K.N.); nwkangkr@snu.ac.kr (N.-W.K.); oek614@snu.ac.kr (E.K.O.); jdm721@snu.ac.kr (D.M.J.); ddkim@snu.ac.kr (D.-D.K.); 2 College of Pharmacy, Chungnam National University, Daejeon 34134, Korea; jaeyoung@cnu.ac.kr; 3 College of Pharmacy, Chung-Ang University, Seoul 06974, Korea; hyunjungkim@cau.ac.kr

**Keywords:** N-degron pathway, N-terminal asparagine amidohydrolase 1, substrate specificity

## Abstract

The N-degron pathway is a proteolytic system in which a single N-terminal amino acid acts as a determinant of protein degradation. Especially, degradation signaling of N-terminal asparagine (Nt-Asn) in eukaryotes is initiated from its deamidation by N-terminal asparagine amidohydrolase 1 (NTAN1) into aspartate. Here, we have elucidated structural principles of deamidation by human NTAN1. NTAN1 adopts the characteristic scaffold of CNF1/YfiH-like cysteine hydrolases that features an α-β-β sandwich structure and a catalytic triad comprising Cys, His, and Ser. In vitro deamidation assays using model peptide substrates with varying lengths and sequences showed that NTAN1 prefers hydrophobic residues at the second-position. The structures of NTAN1-peptide complexes further revealed that the recognition of Nt-Asn is sufficiently organized to produce high specificity, and the side chain of the second-position residue is accommodated in a hydrophobic pocket adjacent to the active site of NTAN1. Collectively, our structural and biochemical analyses of the substrate specificity of NTAN1 contribute to understanding the structural basis of all three amidases in the eukaryotic N-degron pathway.

## 1. Introduction

Protein degradation plays crucial roles in regulating half-lives and functions of proteins. Additionally, protein degradation is a mechanism of protein quality control preventing the accumulation of misfolded, damaged, and aggregated proteins induced by cell stresses, which could lead to some human diseases [1,2,3,4,5,6,7,8,9,10,11,12,13,14]. In eukaryotic cells, protein degradations are mediated by two major pathways, the ubiquitin-proteasome system (UPS) and lysosomal proteolysis. Proteins destined for degradation contain signals leading to subsequent proteolytic machineries and the signals are termed degrons. Especially, exposure or modification of N-terminal amino acid residues can mediate protein degradation and the resultant proteolytic procedure is called the N-degron pathway (formerly the N-end rule pathway) [15,16,17]. Recently, all 20 N-terminal amino acids have been reported to induce proteolysis via the arginylation, acetylation, or proline branches of the N-degron pathway in *Saccharomyces cerevisiae* [18,19,20].

In the mammalian Arg/N-degron pathway, destabilizing N-terminal residues can be classified into primary, secondary, and tertiary degron [21]. Two tertiary degrons, Asn and Gln, are hydrolyzed into the secondary degron Asp and Glu by N-terminal asparagine amidohydrolase 1 (NTAN1) [22,23,24,25] and N-terminal glutamine amidohydrolase 1 (NTAQ1) [26,27,28], respectively. The other tertiary degron, N-terminal cysteine (Nt-Cys), is oxidized into a secondary destabilizing residue [29,30,31], and recently a family of cysteine oxidases was reported to catalyze the oxidation of Nt-Cys in plants [32]. Each of these three secondary degrons can be conjugated with the amino acid Arg by ATE1 arginyltransferase, which generates the type-1 primary degron Nt-Arg [18,33,34]. Nt-Arg, other type-1 degrons (Lys and His), and type-2 degrons (Trp, Phe, Tyr, Leu, and Ile) are recognized and directly bound by specific recognition components, called N-recognins including the mammalian Ubr1, Ubr2, Ubr4, and Ubr5 E3 ligases [25,33,35,36,37,38,39,40]. These ligases facilitate substrate ubiquitination and proteasomal degradation via UPS [33,37,41]. Recent studies have shown that Nt-Arg can also induce autophagic targeting through its binding to the N-recognin p62/SQTSM-1/Sequestosome-1, leading to autophagosomal targeting and lysosomal degradation [42,43].

Nt-Asn and Nt-Gln can act as tertiary degrons in yeast and plants, as well as in mammals [18,21,44,45]. In yeast, both Nt-Asn and Nt-Gln are deamidated by N-terminal amidase 1 (yNta1), which has the structural fold of the nitrilase superfamily [46,47]. In multicellular eukaryotes, Nt-Asn and Nt-Gln are deamidated by *NTAN1*-encoded Nt^N^-amidase and *NTAQ1*-encoded Nt^Q^-amidase, respectively [22,23,24,25,26,27,28]. We have previously determined the crystal structure of human NTAQ1 and proposed a Cys protease-like reaction mechanism [27]. Although yNta1, NTAQ1, and NTAN1 do appear to share catalytic functions commonly involving a conserved Cys residue critical for the deamidation activity, they exhibit low sequence similarity to each other. This implies that they have different evolutionary origins and were independently recruited to the N-degron pathway [27,41,47,48]. The physiological importance of Nt-Asn/Nt-Gln deamidation was first demonstrated by NTAN1-knockout mice exhibiting impaired memory, learning, and social behavior [25,49]. However, the structural basis of NTAN1 has not yet been established.

In this study, the crystal structure of NTAN1, the last veiled structure among three amidases in the N-degron pathway, was elucidated. And we explain unexpected substrate specificities of NTAN1 based on structural and biochemical analyses. Finally, our studies provide a full structural comparison among all three amidases in the N-degron pathway.

## 2. Materials and Methods

### 2.1. Cloning, Protein Expression, and Purification

We synthesized codon-optimized NTAN1 gene of which sequence information was provided by Georgiou et al. for protein expression in *Escherichia coli*. The gene for full-length NTAN1 was cloned into pET-21a(+) (Novagen, Darmstadt, Germany) between Nde1 and Xho1 restriction sites to have C-terminal hexahistidine tag. NTAN1 was overexpressed in the Rosetta 2(DE3), *E*. *coli* strain (Novagen, Darmstadt, Germany). The transformed cells were grown in Luria-Bertani media containing ampicillin at 37 °C until the optical density at 600 nm reached 0.5 and protein expression was induced by 0.5 mM isopropyl β-d-1-thiogalactopyranoside. The cells were shake-incubated for additional for 16 h at 18 °C and harvested by centrifugation at 6000 × *g* for 10 min (Figure S1). The harvested cells were resuspended in a buffer containing 500 mM NaCl, 20 mM Tris-HCl (pH 7.5), 35 mM imidazole, and 1 mM phenylmethylsulfonyl fluoride and subsequently lysed by sonication. Lysed cells were centrifuged at 35,000 × *g* for 1 h. The resultant supernatant was filtered with 0.45 μm filter device (Sartorius, Göttingen, Germany) and loaded onto a 5-mL HiTrap Chelating HP column (GE Healthcare, Chicago, IL, USA) which had been pre-charged with Ni^2+^ and equilibrated with a buffer containing 500 mM NaCl, 20 mM Tris-HCl (pH 7.5), and 35 mM imidazole. After nonspecifically bound proteins were eluted, the retained proteins were eluted by addition of an increasing gradient of a buffer containing 500 mM NaCl, 20 mM Tris-HCl (pH 7.5), and 1 M imidazole. The proteins were loaded onto a HiPrep Desalting 26/10 column (GE Healthcare, Chicago, IL, USA) and eluted with a buffer containing 50 mM NaCl and 20 mM Tris-HCl (pH 7.5) The eluates were loaded onto a HiTrap 5-mL Q HP column (GE Healthcare, Chicago, IL, USA) which had been equilibrated with a buffer containing 50 mM NaCl and 20 mM Tris-HCl (pH 7.5). After nonspecifically bound proteins were eluted, the retained proteins were eluted with addition of increasing gradient of a buffer containing 1 M NaCl and 20 mM Tris-HCl (pH 7.5). Finally, the proteins were concentrated, loaded onto a HiLoad 16/600 Superdex 200 pg column (GE Healthcare, Chicago, IL, USA), and eluted with a buffer containing 200 mM NaCl and 10 mM HEPES-NaOH (pH 7.0). Purity of the protein fractions was checked by sodium dodecyl sulfate-polyacrylamide gel electrophoresis after each purification step.

For the selenomethionine-derived protein, NTAN1 was overexpressed in B834(DE3), an *E. coli* strain. The cells were cultured in the media containing M9, minimal salts (Sigma-Aldrich, Darmstadt, Germany) and amino acid mix containing L-selenomethionine. The protein was expressed and purified as for native NTAN1.

### 2.2. Mutagenesis

NTAN1 mutants were constructed using QuikChange Ⅱ Site-Directed Mutagenesis Kit (Agilent Technologies, Santa Clara, CA, USA). NTAN1 mutants, Q51A, S69A, T73A, T74A, C75A, H92A, S254A, T255A, and E260A were generated. The mutants were expressed and purified as for NTAN1.

### 2.3. Crystallography

Purified NTAN1 was crystallized at 22 °C using the sitting and hanging drop vapor diffusion method for screening and optimization, respectively, with 1 µL protein and 1 µL crystallization solution. Initial crystals of native NTAN1 were grown under a commercial crystallization screening condition with 0.15 M potassium phosphate dibasic and 20% (w/v) polyethylene glycol (PEG) 3350 (PEG/Ion Screen; Hampton Research, Aliso Viejo, CA, USA). The crystallization condition was further optimized for x-ray data collection. The crystals were cryoprotected with reservoir solution supplemented with 12.5% of glycerol and flash-cooled in a nitrogen gas stream at 100 K. Diffraction data were collected at the synchrotron beamline 5C of the Pohang Light Source, Republic of Korea, and processed at 1.90 Å resolution using the HKL2000 program suite [50]. For the NTAN1-substrate peptide complex structures, crystals of NTAN1 C75S were soaked into the reservoir solution containing 10 mM synthetic peptides (NLAAR, NFAAR, NAAAR, and NRAAR) for 16 hr before x-ray diffraction data were collected.

The initial crystal of selenomethionine-derived NTAN1 was grown under commercial crystallization screening condition with 0.2 M sodium malonate (pH 5.0) and 20% (w/v) PEG 3350 (PEG/Ion Screen; Hampton Research, Aliso Viejo, CA, USA) at 22 °C. The crystals were further optimized for x-ray diffraction experiments. Single-wavelength anomalous diffraction (SAD) data from the optimized crystals were collected at the anomalous peak wavelength 0.97928 Å and processed at 2.85 Å resolution. An initial model was built by AutoSol program in the *PHENIX* software package [51] using the SAD method with a high-resolution limit of 3.2 Å for phasing and further improved by density modification using the auto-model building program *Resolve* [52]. Then, the model was used as a template for molecular replacement method using the *Phaser* program [53] to obtain phases for the diffraction data collected from the native NTAN1 crystals. Model building, refinement, and validation for crystal structures were implemented by the WinCoot [54], phenix.refine [55], and MolProbity [56] programs, respectively. Data collection and refinement statistics are summarized in Table S1.

### 2.4. Deamidation Assay Quantified by LC-MS Analysis

Peptides with Nt-Asn which mimic potential substrates of NTAN1 were synthesized: NRA, NRAA, NRAAA, NRQVA, NRQVAA, NRQVAAA, NFAAR, NLAAR, NRAAR, NAAAR, NNAAR, NPAAR, NGAAR, and NDAAR. Non-Nt-Asn peptides, QRAAA and VRAAA, were also synthesized. NTAN1 (at concentrations of 0.5 μM, 0.16 μM, or 0.04 μM) and the peptides (at a concentration of 100 μM) were mixed and incubated at 37 °C in a buffer containing 200 mM NaCl and 10 mM HEPES-NaOH (pH 7.0). The reactants sampled at various incubation time points (of 1, 2, 5, 10, and 20 min) were heat-inactivated at 95 °C for 10 min to stop further reaction. Subsequently, the samples were centrifuged at 20,000 × *g* and diluted 1/50 times with double-deionized water (DW) for liquid chromatography-mass spectrometry (LC-MS) analysis. The deamidation coefficient of peptides catalyzed by NTAN1 was assessed by the slope of exponential curve drawn from the concentration of the substrate over incubation time.

The peptides were quantified by an LC-MS system (1260 Infinity/6430 Triple Quad, Agilent Technologies) equipped with a binary pump (G1312B), a thermostated column compartment (G1316C), a thermostat (G1330B), and an autosampler (G1367E). Chromatographic separation was carried out by injecting an aliquot (2 µL) of analytical sample into a Kinetex C18 column (2.6 μm, 100 Å, 100 × 4.6 mm; Phenomenex, Torrance, CA, USA) with a C18 guard column (4 × 2.0 mm; Phenomenex, Torrance, CA, USA). The mobile phase consisted of acetonitrile and DW (30:70, v/v) with 0.2% (v/v) formic acid. The elution was conducted under an isocratic condition at a flow rate of 0.4 mL/min (total run time: 2.5 min). Mass spectrometric detection of peptides was achieved using the selected ion monitoring (SIM) mode with positive electrospray ionization (ESI). The ESI source settings were manually optimized and the set-up values were as follows: gas temperature: 350 °C; gas flow: 11 L/min; nebulizer pressure: 30 psi (nitrogen); capillary voltage: 3,000 V. The dwell time was 50 ms and the mass filter was set at unit resolution. The optimized mass-to-charge ratio (*m*/*z*), fragmentor voltage (V), and retention time of each peptide are listed in Table S2. The data acquisition and processing were performed by using MassHunter Workstation Software Quantitative Analysis (Version B.05.00; Agilent Technologies, Santa Clara, CA, USA).

### 2.5. Surface Plasmon Resonance (SPR)

SPR binding assays were performed using a carboxymethyl dextran (CM5) sensor chip on a Biacore T200 instrument (GE Healthcare, Chicago, IL, USA). The amine coupling for ligand immobilization was performed at a flow rate of 5 μL/min. The chip was activated with a mixture of *N*-hydroxysuccinimide and *N*-(3-Dimethylaminopropyl)-*N*′-ethylcarbodiimide hydrochloride at a ratio of 1:1 for 400 s. Then, 0.08 mg/mL NTAN1 C75S were diluted in 10 mM sodium acetate (pH 5.5) and injected until the immobilization level reached at 3000 RU. The remaining activated carboxyl groups were deactivated with 1 M ethanolamine at pH 8.5 for 400 s. The multi-cycle analysis was performed at a flow rate of 30 μL/min. Peptides at concentrations of 1.95, 3.91, 7.81, 15.63, 31.25, and 62.50 μM in the running buffer (150 mM NaCl, 10 mM HEPES-NaOH (pH 7.2), 0.1 mM TCEP, and 0.001% polysorbate 20) were injected over the chip for 120 s, followed by dissociation for 600 s in a separate analysis cycle. The sensor chip surface was regenerated with 5 mM NaOH between each cycle. Data were fitted using the simple bimolecular 1:1 Langmuir isotherm binding model. The equilibrium dissociation constant (K_D_) was determined using Biacore T200 evaluation software 3.0 (GE Healthcare, Chicago, IL, USA).

### 2.6. Statistical Analysis

Statistical analysis was performed using Bonferroni’s multiple comparison test. All graphs are presented as the mean ± standard deviation of three independent experiments. Differences with *p* value < 0.05 were considered statistically significant (*** *p* < 0.005; * *p* < 0.05).

### 2.7. Data Availability

The atomic coordinates and structural factor data from this research have been deposited to the Protein Data Bank (www.wwpdb.org) and assigned the PDB IDs, 6A0E (NTAN1 wild-type), 6A0I (NTAN1 C75S mutant), 6A0H (NTAN1 C75S mutant in complex with the NLAAR peptide), and 6A0F (NTAN1 C75S mutant in complex with the NFAAR peptide).

## 3. Results

### 3.1. NTAN1 Structurally Belongs to the CNF1/YfiH-Like Cysteine Hydrolase Family

**Figure 1:**
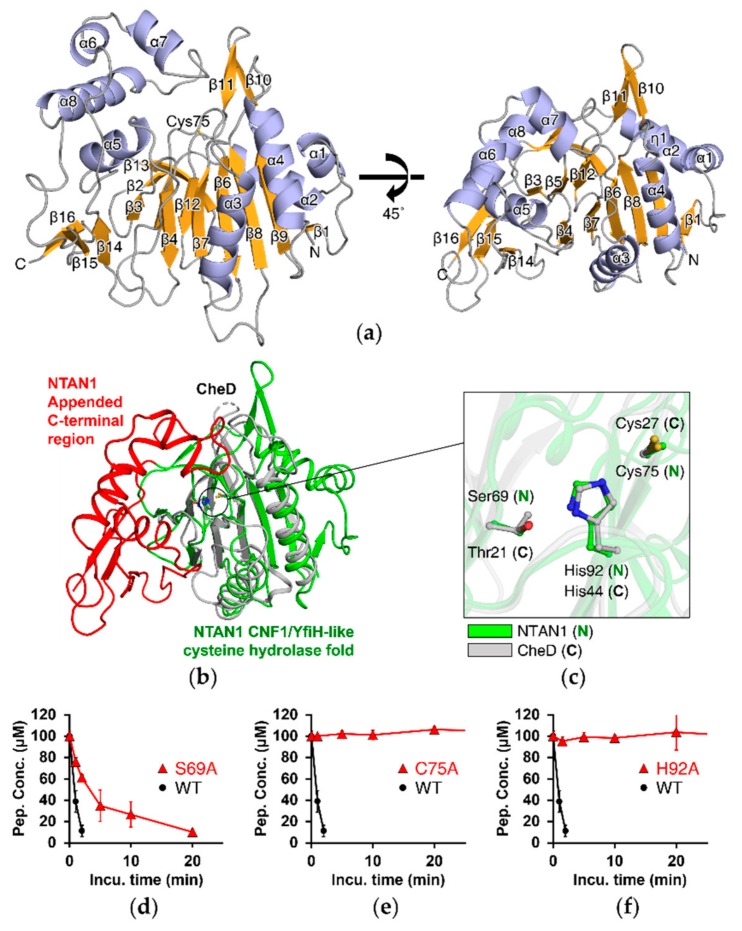
NTAN1 has a CNF1/YfiH-like cysteine hydrolase fold with an appended C-terminal region. (**a**) The α-β-β sandwich structure of NTAN1. Helices (eight α-helices, α1–α8; and one 3_10_-helix, η1), β-strands (β1–β16), and loops are shown as cartoon representation colored in light blue, bright orange, and gray, respectively. The NTAN1 structure on the left-hand side is rotated vertically by 45° on the right-hand side to effectively display the α-β-β sandwich structure and the catalytically crucial Cys75 in the active site. (**b**) Superposition of NTAN1 with CheD from *Thermotoga maritima*. CNF1/YfiH-like cysteine hydrolase fold and appended C-terminal region of NTAN1 are colored in green and red, respectively. CheD is colored in gray. The active sites of both proteins are marked with a black circle. (**c**) Close-up view of the superposed active sites of NTAN1 and CheD. Catalytic triads of NTAN1 (Cys75/His92/Ser69) and CheD (Cys27/His44/Thr21) are shown as green and gray ball-and-stick models, respectively. Nitrogen, oxygen, and sulfur atoms are colored in blue, red, and yellow, respectively. (**d**–**f**) Deamidation by Ala-mutants of NTAN1 catalytic triad. Concentrations of the NRAAA peptide are plotted against incubation times with NTAN1 (wild type, WT) and the mutants, S69A (**d**), C75A (**e**), and H92A (**f**). Error bars indicate standard-deviation values of three independent experiments.

To elucidate the structural principles underlying deamidation of Nt-Asn, we purified wild-type NTAN1 with a C-terminal hexahistidine-tag and determined its crystal structure at 1.95 Å resolution (Table S1). The initial phase was determined by the single-wavelength anomalous dispersion method with selenomethionine-derived crystals of NTAN1. The space group of the crystal was P2_1_2_1_2_1_ and the asymmetric unit contained two NTAN1 molecules. We resolved all residues except for the initial methionine and the four C-terminal residues connected to the hexahistidine-tag. This 310-residue enzyme comprises eight α-helices, one 3_10_-helix, and sixteen β-strands, and adopts an α-β-β sandwich structure made of two mixed β-sheets and flanking helices (Figure 1a).

The primary sequence of NTAN1 showed no similarity to known proteins in either eukaryotes or prokaryotes. However, analysis using the *DALI* web server [57] revealed that the overall structural folding of NTAN1 showed similarity to those of some CNF1/YfiH-like cysteine hydrolases that mediate deamidation of specific internal Gln residues of their substrates [58,59,60,61]. Among these, NTAN1 showed the highest similarity (Z-score = 13.6, sequence identity = 17%) to *Thermotoga maritima* glutamine deamidase CheD (PDB ID: 2F9Z), which hydrolyzes specific Gln residues on methyl-accepting chemotaxis proteins (MCPs), the receptors involved in transmission of chemotaxis signals [59]. High three-dimensional similarities were also found with the *Shigella flexneri* polyphenol oxidase YfiH (Z-score = 7.3, sequence identity = 5%), the *Burkholderia pseudomallei* lethal factor BLF1/BPSL1549 (Z-score = 5.4, sequence identity = 4%) that catalyzes the site-specific deamidation of Gln399 of the eukaryotic translation initiation factor elF4A [61], and the *E*. *coli* cytotoxic necrotizing factor CNF1 (Z-score = 4.8, sequence identity = 14%) that catalyzes the site-specific deamidation of Gln residues in RhoA, Rac, and Cdc42 [58]. Superposing the structural folds of these deamidase enzymes revealed that their three-dimensional similarity was most prominent in the β-sandwich domain.

Superposing the NTAN1 and CheD structures clearly showed the overlapping β-sandwich structure, and the conserved catalytic triad composed of Cys75, His92, and Ser69 of NTAN1 (Figure 1b,c). Cys75 and His92 of NTAN1 were strictly conserved, while Ser69 of NTAN1 was more diverse among the CNF1/YfiH-like cysteine hydrolase family; CheD and BLF1 have Thr, and CNF1 has a backbone carbonyl oxygen, instead of Ser (Figure S2). Despite the similarities of the folded structures, we also observed structural differences between NTAN1 and other CNF1/YfiH-like cysteine hydrolases. For example, NTAN1 lacked the Tyr residue that interacts with the backbone carbonyl of the catalytic Cys in BLF1/CNF1, and a zinc ion that is coordinated at the active site of YfiH. The most outstanding feature of NTAN1 is the appended C-terminal region that contributes to its specificity for Nt-Asn, compared with other CNF1/YfiH-like cysteine hydrolase family members (Figure 1b).

### 3.2. Deamidation Activity of NTAN1 Is Mediated by a Canonical Catalytic Triad

**Figure 2:**
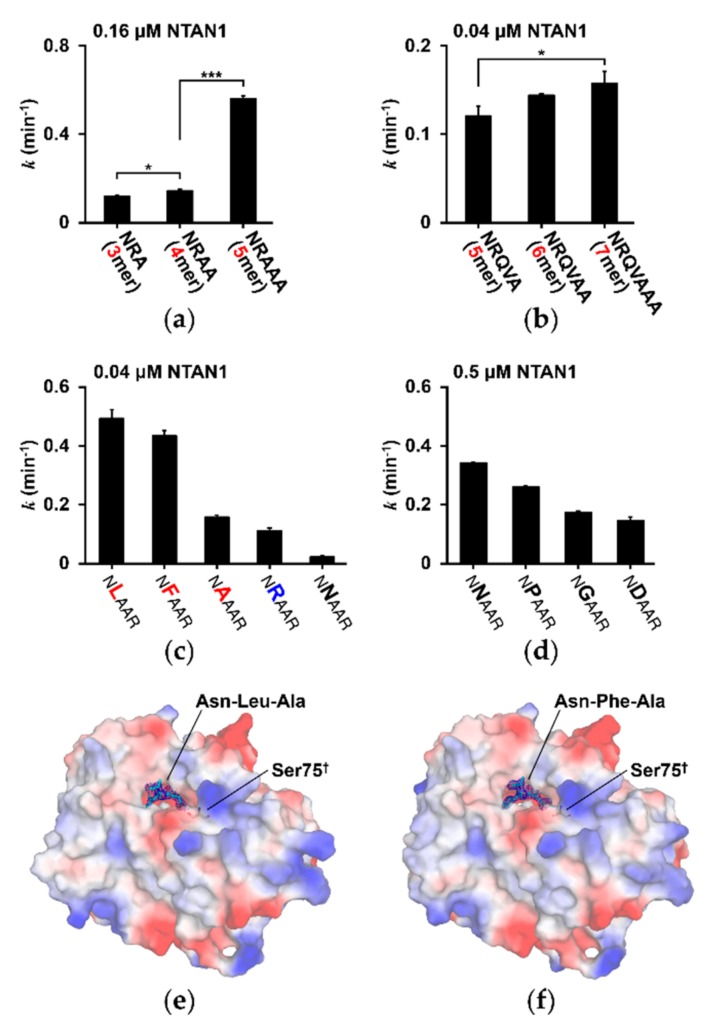
NTAN1 shows different deamidation rates on various Nt-Asn peptides. (**a**) Deamidation rates of NRA, NRAA, and NRAAA peptides for 0.16 μM NTAN1. Deamidation coefficient was obtained from the slope when the logarithmic concentrations of the substrates were plotted against incubation times. *p* values were determined using Bonferroni’s multiple comparison test; *** *p* < 0.005, * *p* < 0.05). Error bars indicate standard-deviation values of three independent experiments. (**b**) Deamidation rate of NRQVA, NRQVAA, and NRQVAAA peptides for 0.04 μM NTAN1. (**c**) Deamidation rates of NXAAR peptides measured with 0.04 μM NTAN1, where the second-position X represents Leu (L), Phe (F), Ala (A), Arg (R), and Asn (N). The hydrophobic and basic residues in second-position are labeled in red and blue, respectively. (**d**) Deamidation rates of NXAAR peptides measured with 0.5 μM NTAN1, where the second-position X represents Asn (N), Pro (P), Gly (G), and Asp (D). (**e**,**f**) Electrostatic surface of NTAN1 C75S mutant structures in complex with NLAAR (**e**) and NFAAR (**f**) pentapeptides. Catalytic Cys75 residues mutated to Ser75 are labeled with daggers. The positively, negatively, and neutrally charged surfaces are colored in blue, red, and white, respectively. Three amino acid residues with definitive electron density from the N-terminus of the pentapeptides are shown as stick models and colored in cyan and the electron densities of *mFo*-*DFc* omit maps are represented by blue meshes at a 3.0σ contour level.

In the CNF1/YfiH-like cysteine hydrolase family, residues comprising the Cys/His/Ser catalytic triad play critical roles in the deamidation reaction. During catalysis, Cys functions as a nucleophile using its thiol, His deprotonates the Cys thiol, and Ser orients His [62]. To characterize the catalytic activity of NTAN1, we developed an in vitro deamidation assay using mass-spectrometric analysis with Nt-Asn peptides (Figure S3). Because no in vivo substrates of NTAN1 have been reported to date, Nt-Asn peptides of varying lengths (3–7 mers) were synthesized, with the second-position residue fixed with Arg to maintain water solubility. The Nt-Asn peptides (NRA, NRAA, and NRAAA) were mixed with 0.16 μM bacterially purified NTAN1 (Figure 2a).

Following deamidation into Nt-Asp peptides, the amounts of unprocessed Nt-Asn peptides were measured using combined liquid chromatography and mass spectrometry. To measure deamidation activities with longer peptides, 0.04 μM NTAN1 was mixed with modified Nt-Asn peptides (NRQVA, NRQVAA, and NRQVAAA to avoid synthetic difficulty of repeating residues) because these peptides were deamidated too rapidly with 0.16 μM NTAN1 (Figure 2b). The results showed that NTAN1 more efficiently hydrolyzed Nt-Asn in longer peptides compared with shorter ones. The most significant increase in deamidation activity was observed when the length of the substrate peptides was increased from 4 mers to 5 mers by approximately threefold, and this demonstrates the effect of substrate peptide length on the catalytic activity of NTAN1.

To further elucidate the contribution of the catalytic triad of NTAN1, we implemented in vitro deamidation assays for wild-type NTAN1 and respective mutants involving catalytic triad (C75A, H92A, and S69A) with NRAAA as a substrate peptide. The deamidation activities of NTAN1 C75A and H92A mutants were completely abolished, and the NTAN1 S69A mutant exhibited an approximately 5-fold lower deamidation activity than wild-type NTAN1 (Figure 1d–f). Interestingly, Cys75, His92, and Ser69 of human NTAN1 are strictly conserved among NTAN1 homologs from various eukaryotes, which consistently implies crucial roles of the catalytic triad (Figure S4).

### 3.3. Substrate Specificity of NTAN1 Is Affected by the Second-Position Residue of Substrates

To gain deeper insights into the substrate specificity of NTAN1, we next examined the deamination activities of NTAN1 with NXAAR substrate peptides containing various representative second-position residues (where X = Leu, Phe, Ala, Arg, Asn, Pro, Gly, and Asp). The enzymatic activity of NTAN1 differed remarkably, depending on the identity of the second-position residue (Figure 2c,d). NTAN1 most efficiently deamidated NLAAR, NFAAR, and NAAAR peptides containing hydrophobic amino acid residues at the second-position. NTAN1 also exhibited moderate deamidation activity for the NRAAR peptide containing a basic amino acid residue at the second-position. In contrast, low deamidation activities were detected with NNAAR, NPAAR, NGAAR, and NDAAR peptides containing polar, cyclic, smallest, and acidic amino acid residues, respectively, at the second-position. These results suggested that NTAN1 preferentially recognizes the Nt-Asn residue followed by hydrophobic amino acid residues at the second-position, such as Leu, Phe, and Ala.

### 3.4. Substrate-Recognition Mode of NTAN1 to Nt-Asn

We further sought to elucidate the structural principles underlying the recognition of Nt-Asn by determining crystal structures of NTAN1 in complex with various Nt-Asn pentapeptides. Since substrate peptides may be converted into product peptides and dissociated from NTAN1, we generated an NTAN1 C75S mutant to fix substrate peptides in the crystal form. The crystal structure of NTAN1 C75S exhibited no noticeable changes, compared with wild-type NTAN1, with C_α_ root-mean-square deviation of 0.23 Å (Figure S5). We therefore soaked the crystals of NTAN1 C75S in crystallization solutions containing NLAAR, NFAAR, NAAAR, or NRAAR. Among these, we were able to resolve the crystal structures of NTAN1 C75S in complex with either NLAAR or NFAAR (Figure 2e,f). It was consistent with our SPR experiments showing that the binding affinity of NLAAR to NTAN1 C75S (K_D_ = 26.84 nM) is higher than that of NAAAR (K_D_ = 4.67 μM). With the assay results involving various length of peptides, we expected to observe the interaction mode of NTAN1 up to the 4th or 5th residues of the peptides, but could confidently model only three N-terminal residues of NLAAR and NFAAR in the NTAN1 complex structures.

In the analysis of binding modes between NTAN1 C75S and peptides, we carefully analyzed two molecules in the asymmetric unit to find all possible binding modes from our complex structural models at 3.2 Å (for NLAAR) and 2.4 Å (for NFAAR) resolutions using *PISA* and *LigPlot^+^* [63,64]. The structures showed that NTAN1 recognizes Nt-Asn via several hydrogen bonds. The N-terminal amine of Nt-Asn formed a salt bridge with the side chain of Glu260 in the appended C-terminal region, and hydrogen bonds with the side-chain oxygen of Thr73 and Thr255 (Figure S6). The side chain of Nt-Asn cooperatively interacted with NTAN1 by forming hydrogen bonds with Asp71, Thr 73, Thr74, Thr255, and Ser75 (equivalent to Cys75 in wild-type NTAN1). The backbone carbonyl of Nt-Asn formed hydrogen bonds with Thr255 and is seemed to interact with Ser254.

**Figure 3:**
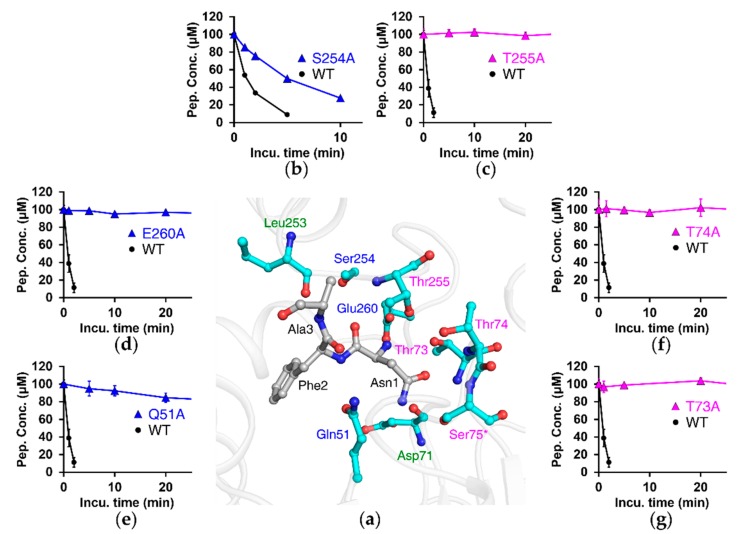
Residues interacting with substrate peptide through hydrogen bonds or salt bridges are crucial in catalytic activity of NTAN1. (**a**) Structure of NTAN1 C75S mutant in complex with the NFAAR pentapeptide at the active site. NTAN1 residues interacting with the peptide through hydrogen bonds or salt bridges are shown in cyan ball-and-stick model (Ser75 mutated from catalytic Cys75 is labeled with an asterisk). Thr73, Thr74, and Thr255 interact with the peptide via both their side chains and backbones, and are labeled in magenta. Gln51, Ser254, and Glu260 interact with the peptide via only side chains, and are labeled in blue. Asp71 and Leu253 interact with the peptide via only backbones, and are labeled in green. The bound peptide is colored in gray. Nitrogen and oxygen atoms are colored in blue and red, respectively. (**b**–**g**) Deamidation activities by Ala-mutants of NTAN1 residues described in (**a**). Concentrations of the NRAAA peptide are plotted against incubation times with NTAN1 WT and mutants, S254A (**b**), T255A (**c**), E260A (**d**), Q51A (**e**), T74A (**f**), and T73A (**g**). Error bars indicate standard-deviation values of three independent experiments.

Next, we assessed the importance of these residues by generating Ala-substituted mutants (Figure 3). In vitro deamidation assays with NRAAA showed that NTAN1 lost its deamidation activity by mutation of Glu260, which otherwise interacts with the N-terminal amine of Nt-Asn. Enzymatic activity was also abolished when the residues forming hydrogen bonds with Nt-Asn were mutated (T73A, T74A, and T255A). Furthermore, mutation of Ser254, which could form a hydrogen bond with the backbone carbonyl of Nt-Asn, exhibited a threefold lower deamidation rate than wild-type NTAN1. Because Thr73, Thr74, Ser254, Thr255, and Glu260 form interactions with Nt-Asn, but are not members of the catalytic triad, these results showed that the recognition of Nt-Asn is critical for the deamidation activity of NTAN1.

### 3.5. NTAN1-Peptide Complex Structures Also Reveal the Importance of Second-Position and, to a Lesser Degree, Third-Position Amino Acid of Substrates

**Figure 4:**
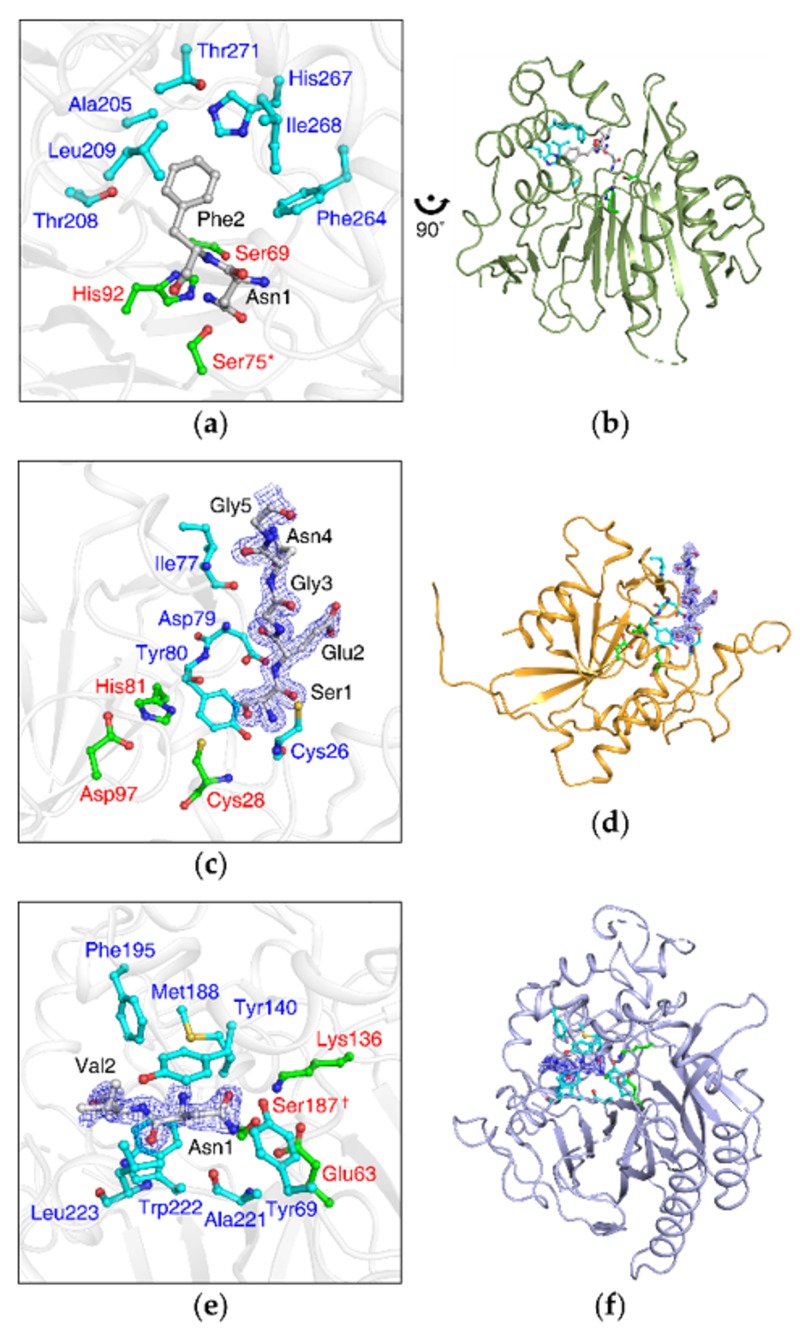
Crystal structures of NTAN1, NTAQ1, and yNta1 exhibit different substrate-recognition modes. (**a**) NTAN1 residues accommodating the second-position residue of substrates. Catalytic triad (Cys75/His92/Ser69, Cys75 is mutated to Ser75 in the structure and labeled with an asterisk) and the residues interacting with the second-position Phe in the NTAN1 C75S–NFAAR complex are colored in green and cyan, respectively. The bound peptide is colored in gray. Nitrogen and oxygen atoms are colored in blue and red, respectively. (**b**) Overall structure of NTAN1. Zoom-out view of (**a**) rotated 90° anticlockwise. (**c**) NTAQ1 structure in complex with a substrate-mimicking peptide (PDB code: 4W79). Catalytic triad (Cys28/His81/Asp97) and the residues forming specific hydrogen bonds with the substrate-mimicking N-terminus of an adjacent NTAQ1 molecule are colored in green and cyan, respectively. The substrate-mimicking N-terminus is colored in gray, and its electron-density map is represented by blue mesh at a 1.5σ contour level. Sulfur atoms are colored in yellow. (**d**) Overall structure of NTAQ1. Zoom-out view of (**c**). (**e**) Structure of yNta1 C187S mutant in complex with NV dipeptide (PDB ID: 5K62). Catalytic triad (Cys187/Glu63/Lys136, Cys187 is mutated to Ser187 in the structure and labeled with a dagger) and the residues interacting with the NV dipeptide are colored in green and cyan, respectively. NV dipeptide is colored in gray, and its electron-density maps are represented by blue mesh at a 1.5σ contour level. (**f**) Overall structure of yNta1. Zoom-out view of (**e**).

Our structural analyses also revealed that NTAN1 contains a pocket comprising the side chains of Ala205, Thr208, Leu209, Phe264, His 267, Ile268, and Thr271 in the appended C-terminal region, adjacent to the catalytic active site (Figure 4a), which mainly stabilized the side chain of the second-position residue Leu2/Phe2 of NLAAR/NFAAR substrate peptides via hydrophobic effects (Figure S6). In addition, the NTAN1 Gln51 amide group formed hydrogen bonds with the peptide backbone of Leu2/Phe2 at the entrance to the pocket. The backbone carbonyl of NTAN1 Leu253 formed a hydrogen bond with the backbone amine of the third-position Ala3 residue (Figure 3a). To validate the contribution of NTAN1 Gln51 to the recognition of the second-position residue, we implemented an in vitro deamidation assay of the NTAN1 Q51A mutant with NRAAA substrate peptide, and observed that the mutant lost most deamidation activity (Figure 3e). These results implied that substrate recognition of NTAN1 is mediated not only by Nt-Asn, but also by the second-position and, to a lesser degree, third-position residues of substrates.

### 3.6. The Appended C-Terminal Region of NTAN1 Is a Key Component in the Recognition of Both Nt-Asn and Subsequent Residues

Our studies revealed the highly organized recognition of Nt-Asn by NTAN1, and an unexpected preference for hydrophobic second-position residues in substrates. NTAN1 shares a structural fold of the CNF1/YfiH-like cysteine hydrolase family, such as CheD, BLF1, and CNF1, which mediates the deamidation of internal Gln residues exposed at the surface of substrate proteins. CheD deamidates internal Gln located in α-helices of MCPs [59], and BLF1/CNF1 deamidates internal Gln located in substrate loop regions [58,61]. Interestingly, the structure of NTAN1 reveals that the appended C-terminal region covers the active site and contribute to the Nt-Asn specificity (Figure 1b). The appended C-terminal region enables NTAN1 to exclude approaches by nonspecific substrates, and to preferentially deamidate Nt-Asn. In addition, the appended C-terminal region contains the residues Ser254, Thr255, and Glu260, which interact with Nt-Asn, and the pocket that accommodates the side chain of second-position residues (Figure 3 and Figure 4a). Taking these observations together, the appended C-terminal region would play a crucial role in substrate specificity of NTAN1.

## 4. Discussion

N-terminal deamidation is a posttranslational modification that has been observed universally in all eukaryotes that have been investigated [28,41]. In yeasts, plants, and mammals, Nt-Asn and Nt-Gln can modulate the stability of protein substrates via their deamidation into Asp and Glu, respectively [28,41]. While yeasts express a single enzyme, yNta1, that mediates deamidation of both Nt-Asn and Nt-Gln, plants and mammals express two distinct enzymes, NTAN1 and NTAQ1, which hydrolyze Nt-Asn and Nt-Gln, respectively [33]. An outstanding question regarding the three enzymes has been how they commonly deamidate tertiary destabilizing residues, and mainly how they accomplish distinctive substrate specificities involving Nt-Asn and Nt-Gln.

Here, we determined the last veiled structure of NTAN1 among the three enzymes and compared our NTAN1 structure with previously determined NTAQ1 and yNta1. NTAN1 has an α-β-β sandwich structural fold with the Cys75/His92/Ser69 catalytic triad (Figure 4a,b). In contrast, NTAQ1 has an α-β-α sandwich structural fold similar to that of protein glutaminases/cysteine proteases, with the Cys28/His81/Asp97 catalytic triad [27] (Figure 4c,d), and yNta1 has an α-β-β-α sandwich structural fold representative of the nitrilase superfamily, with the Cys187/Glu63/Lys136 catalytic triad [47] (Figure 4e,f). The three amidases exhibit different structural folds, and thus have distinctive substrate-recognition modes. While they share the catalytic Cys residues and well-organized interactions with Nt-Asn/Nt-Gln mainly mediated by salt bridges and hydrogen bonds for their respective specificities, remarkable differences are observed in interactions with subsequent residues following Nt-Asn/Nt-Gln.

As described above for NTAN1, peptide backbone and side chain of the substrate second-position residue play critical roles in binding and catalytic activities through specific hydrogen bonds and hydrophobic effects, respectively (Figure 3 and Figure 4a). In the case of NTAQ1, the side chain of second-position residue (Glu2) of the substrate-mimicking peptide extended outward from the active site, and was not recognized by NTAQ1 [27] (Figure 4c). Instead, NTAQ1 formed additional hydrogen bonds with the backbone of the substrate-mimicking peptide up to the fourth residue, which seems to involve more intensive interactions than for NTAN1 and yNta1. As for yNta1, it was shown that both peptide backbone and side chain of the second-position residue of substrates is mainly recognized via hydrophobic effects, and accomplishes its dual specificity for both Nt-Asn and Nt-Gln by distortion of the side chain of Nt-Gln [47] (Figure 4e).

## 5. Conclusions

Based on the structural analyses, all three amidases in the eukaryotic N-degron pathway seem to have been independently recruited via convergent evolution to modulate the metabolic stability of proteins bearing Nt-Asn and Nt-Gln. Our study will shed light on the mechanism (Figure S7) and substrate specificity of NTAN1, and provide valuable clues for related researches including elucidation of physiological substrates of NTAN1 that undergo degradation via the Arg/N-degron pathway.

## Supplementary Materials

The following are available online at https://www.mdpi.com/2218-273X/10/1/163/s1, Figure S1: NTAN1 protein expression by the Rosetta 2(DE3) strain, Figure S2: The catalytic triads of NTAN1 and three other proteins structurally similar to NTAN1, Figure S3: Deamidation of pentapeptides catalyzed by NTAN1, Figure S4: Sequence alignment of NTAN1s from seven representative organisms, Figure S5: Superposition of wild-type NTAN1 and the NTAN1 C75S mutant structures, Figure S6: Substrate-recognition mode of NTAN1, Figure S7: Proposed catalytic mechanism of NTAN1, Table S1: Data collection and refinement statistics of NTAN1 structures, Table S2: Optimized MS parameters and retention times of substrates peptides.
