# Supplementary material for: Structural Analyses on the Deamidation of N-Terminal Asn in the Human N-Degron Pathway
Source: Biomolecules. 2020 Jan 20;10(1):163. doi: 10.3390/biom10010163 (PMC7022378; doi:10.3390/biom10010163)
Supplement: Supplementary file 1 [file biomolecules-10-00163-s001.pdf]

Supplementary Materials

Structural Analyses on the Deamidation of N-Terminal Asn in the Human N-Degron Pathway

Joon Sung Park [1], Jae-Young Lee [2], Yen Thi Kim Nguyen [1], Nae-Won Kang [1], Eun Kyung Oh [1], Dong Man Jang [1], Hyun-Jung Kim [3], Dae-Duk Kim [1] and Byung Woo Han [1,*]

[1] Research Institute of Pharmaceutical Sciences, College of Pharmacy, Seoul National University, Seoul 08826, Korea; wingpjs@snu.ac.kr (J.S.P.); kimyen@snu.ac.kr (Y.T.K.N.); nwkangkr@snu.ac.kr (N.-W.K.); oek614@snu.ac.kr (E.K.O.); jdm721@snu.ac.kr (D.M.J.); ddkim@snu.ac.kr (D.-D.K.)

[2] College of Pharmacy, Chungnam National University, Daejeon 34134, Korea; jaeyoung@cnu.ac.kr

[3] College of Pharmacy, Chung-Ang University, Seoul 06974, Korea; hyunjungkim@cau.ac.kr

* Correspondence: bwhan@snu.ac.kr; Tel.: +82-2-880-7899

Received: 29 November 2019; Accepted: 13 January 2020; Published: date

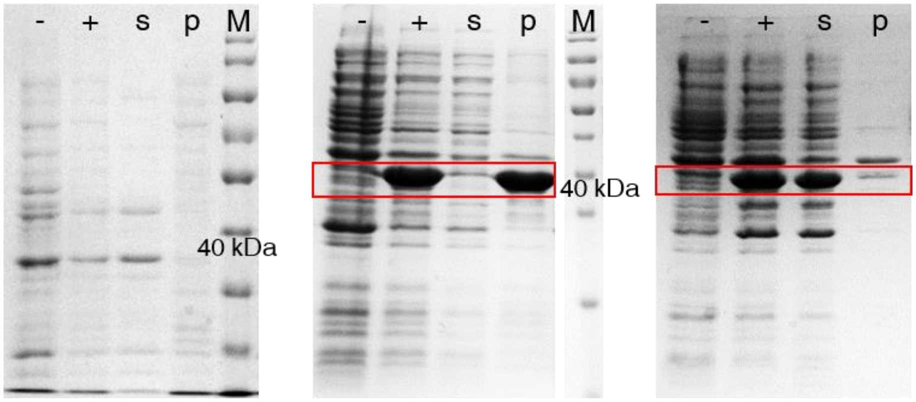

(a) (b) (c)

Figure S1. NTAN1 protein expression by the Rosetta 2(DE3) strain. (**a**) SDS-PAGE analysis of NTAN1 protein expression at 37 °C with wild-type NTAN1 DNA sequences. NTAN1 was rarely expressed and observed. '-' and '+' represent non-induced and IPTG-induced NTAN1 samples. 's' and 'p' represent soluble and insoluble (pelleted) fractions from the IPTG-induced NTAN1 samples, respectively. 'M' represents a protein molecular weight marker (PageRuler™, Thermo Scientific). (**b**) SDS-PAGE analysis of NTAN1 protein expression at 37 °C with the codon-optimized NTAN1 DNA sequences. NTAN1 bands are marked with a red box in the gel. (**c**) SDS-PAGE analysis of NTAN1 protein expression at 18 °C with codon-optimized NTAN1 DNA sequences. NTAN1 bands are marked with a red box in the gel.

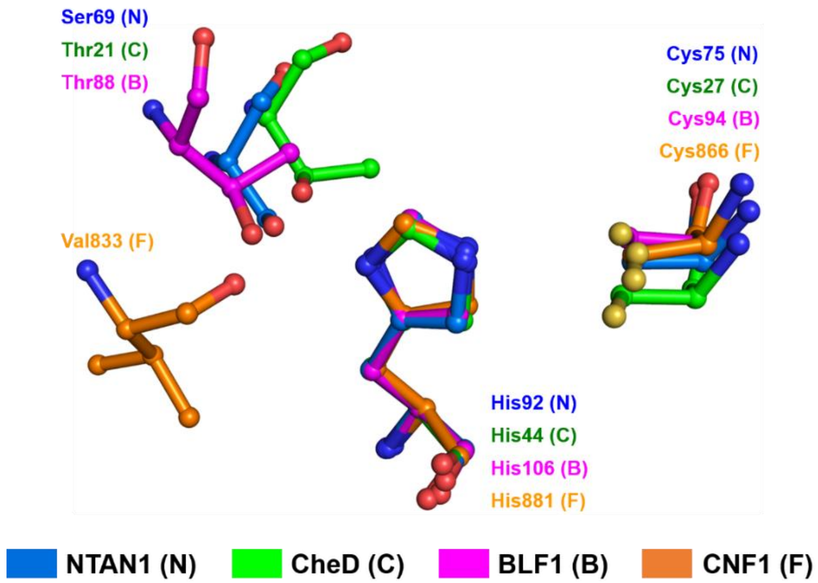

Figure S2. The catalytic triads of NTAN1 and three other proteins structurally similar to NTAN1. The catalytic triad histidines of CheD, BLF1, and CNF1 are superposed on His92 of NTAN1. Carbon atoms of NTAN1, CheD, BLF1, and CNF1 are colored in light blue, green, magenta, and orange, respectively.

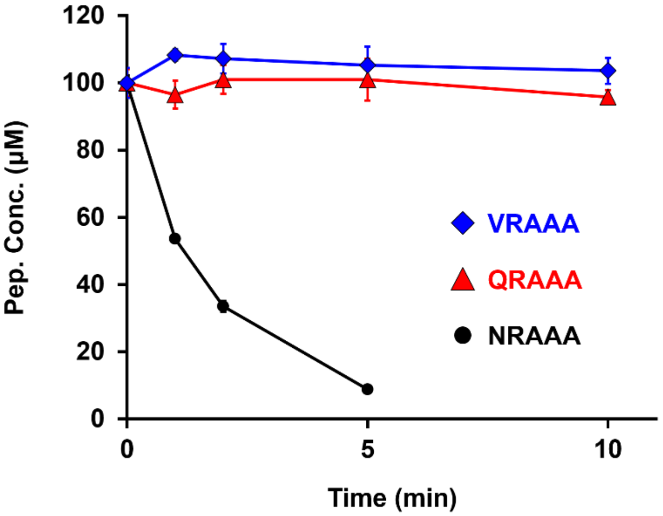

Figure S3. Deamidation of pentapeptides catalyzed by NTAN1. QRAAA and VRAAA pentapeptides were used as controls that do not contain Nt-Asn. The peptides were incubated with NTAN1 and their concentrations were plotted against incubation times. Error bars indicate standard-deviation values of three independent experiments.

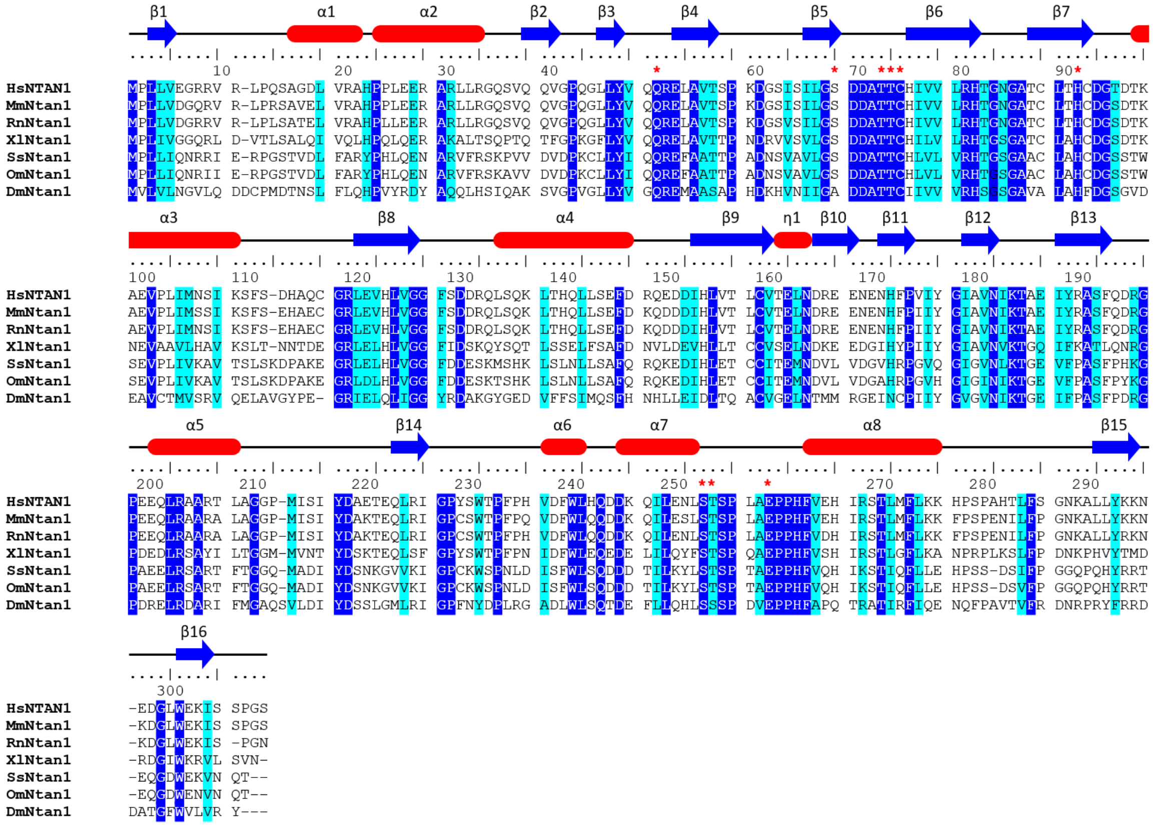

Figure S4. Sequence alignment of NTAN1s from seven representative organisms. Identical and highly conserved residues are marked with blue and cyan boxes, respectively. Secondary structures of human NTAN1 are shown above the sequence numbers. Helices and β-strands are represented as red tubes and blue arrows, respectively. Residues mutated in our study are indicated with red asterisks. Abbreviations are as follows: HsNTAN1, *Homo sapiens* NTAN1; MmNtan1, *Mus musculus* Ntan1; RnNtan1, *Rattus norvegicus* Ntan1; XlNtan1, *Xenopus laevis* Ntan1; SsNtan1, *Salmo salar* Ntan1; OmNtan1, *Oncorhynchus mykiss* Ntan1; DmNtan1, *Drosophila melanogaster* Ntan1.

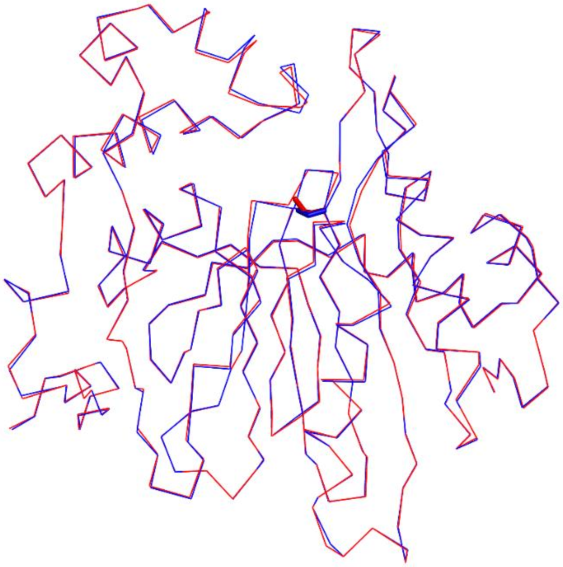

Figure S5. Superposition of wild-type NTAN1 and the NTAN1 C75S mutant structures. Wild-type NTAN1 and the NTAN1 C75S mutant are shown in red- and blue-ribbon representations, respectively.

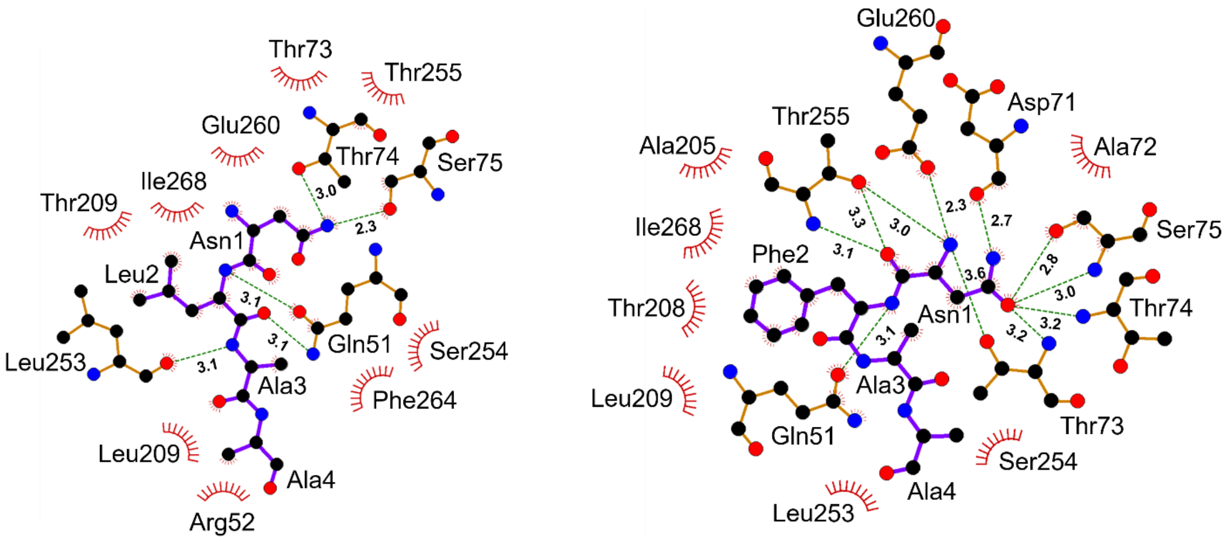

(**a**) (**b**)

Figure S6. Substrate-recognition mode of NTAN1. *LigPlot*+ analyses of bound NLAAR (**a**) and NFAAR (**b**) pentapeptides in the NTAN1 C75S mutant structures.

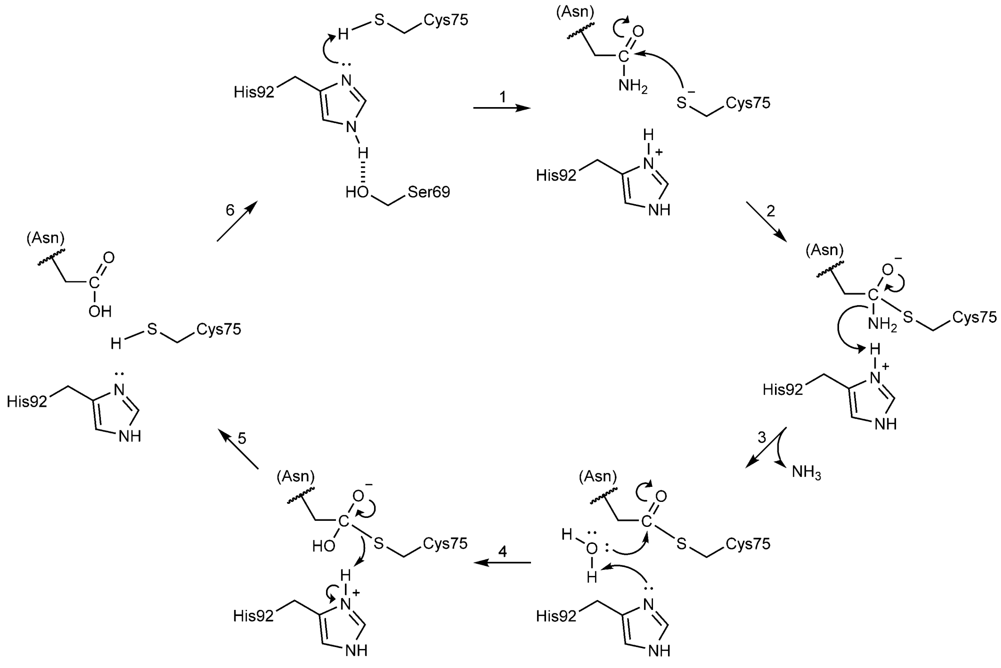

Figure S7. Proposed catalytic mechanism of NTAN1.

Table S1. Data collection and refinement statistics of NTAN1 structures.

	SeMet NTAN1 (SAD)	NTAN1 (PDB ID: 6A0E)	NTAN1 C75S (PDB ID: 6A0I)	NTAN1 C75S-NLAAR (PDB ID: 6A0H)	NTAN1 C75S-NFAAR (PDB ID: 6A0F)
Data collection					
Space group	$P2_12_12_1$	$P2_12_12_1$	$P2_12_12_1$	$P2_12_12_1$	$P2_12_12_1$
Cell dimensions					
a , b, c (Å)	84.86, 85.52, 87.51	83.15, 84.90, 87.20	82.98, 84.34, 87.00	84.00, 85.88, 88.09	84.05, 85.75, 88.30
α, β, γ (°)	90, 90, 90	90, 90, 90	90, 90, 90	90, 90, 90	90, 90, 90
	Peak				
Wavelength (Å)	0.97928	0.97960	0.97960	0.97933	0.97933
Resolution (Å)[a]	50.00 - 2.85 (2.90 - 2.85)	50.00 - 1.95 (1.98 - 1.95)	50.00 - 2.00 (2.03 - 2.00)	50.00 - 3.20 (3.26 - 3.20)	50.00 - 2.40 (2.44 - 2.40)
No. of unique reflections	15317	45885	42029	11141	25940
R_{merge}[a]	0.164 (0.690)	0.091 (0.701)	0.121 (0.602)	0.172 (0.480)	0.092 (0.502)
R_{pim}[a]	0.042 (0.174)	0.037 (0.276)	0.048 (0.247)	0.067 (0.182)	0.035 (0.190)
I/σ(I)[a]	35.0 (8.7)	21.2 (3.2)	16.3 (3.0)	9.7 (3.8)	18.4 (3.7)
Completeness (%)[a]	99.9 (100.0)	100.0 (100.0)	100.0 (100.0)	99.8 (100.0)	99.9 (100.0)
Redundancy[a]	15.7 (16.1)	7.3 (7.3)	7.2 (6.8)	7.6 (7.9)	7.8 (7.9)
Refinement					
Resolution (Å)		30.42 - 1.95	38.53 - 2.00	35.52 - 3.19	30.76 - 2.38
R_{work}/R_{free}[b]		0.175/0.224	0.164/0.211	0.185/0.242	0.196/0.248
No. atoms		5652	5474	4895	5193
Protein		4984	4885	4775	4799
Ligand/ion[c]		75	147	107	176
Water		593	442	13	218
Average B factors (Å^2)		33.0	35.9	48.5	45.2
Protein		31.8	34.5	48.3	44.8
Ligand/ion		54.3	59.7	60.0	59.5
Water		40.7	43.7	32.1	42.5
Ramachandran plot					
Favored (%)		98.02	98.18	98.00	98.02
Allowed (%)		1.98	1.82	2.00	1.98
Outlier (%)		0	0	0	0
R.m.s. deviations					
Bond lengths (Å)		0.002	0.005	0.002	0.002
Bond angles (°)		0.490	0.726	0.509	0.464

[a] Values in parentheses are for the highest-resolution shell.

[b] About 5% of the reflections were excluded from the refinement for R_{free} calculation.

[c] Ligand/Ion includes five glycerol molecules (with hydrogen atoms) and one phosphate molecule (6A0E); 22 glycerol molecules and three phosphate molecules (6A0I); NLAAR peptide, five glycerol molecules, and six phosphate molecules (6A0H); NFAAR peptide, 13 glycerol molecules, and eight glycerol molecules (6A0F). Glycerol and phosphate molecules might be incorporated from crystallization/cryoprotection solutions.

Table S2. Optimized MS parameters and retention times of substrates peptides.

Peptide sequence	Molar mass (g/mol)	Mass-to-charge ratio (*m*/*z*)	Fragmentor voltage (V)	Retention time (min)
NRA	359.39	360.3	120	1.92
NRAA	430.47	431.3	130	1.90
NRAAA	501.55	502.3	120	1.90
NRQVA	586.65	587.3	130	1.91
NRQVAA	657.73	658.4	145	1.91
NRQVAAA	728.81	729.4	170	1.91
NLAAR	543.63	544.4	200	1.92
NFAAR	577.65	578.5	120	1.93
NAAAR	501.55	502.4	170	1.90
NRAAR	586.66	587.5	170	1.85
NPAAR	527.59	528.4	160	1.90
NNAAR	544.57	545.4	120	1.90
NGAAR	487.52	488.3	160	1.90
NDAAR	545.56	546.4	110	1.91
QRAAA	515.28	516.0	130	1.90
VRAAA	486.29	487.4	166	1.91
